# The value of joint peer review between early career researchers and supervisors

**DOI:** 10.1016/j.tcb.2025.03.002

**Published:** 2025-04-03

**Authors:** Viviana Macarelli, Florian T. Merkle

**Affiliations:** 1 Institute of Metabolic Science, University of Cambridge, Cambridge CB2 0QQ, UK; 2 Cambridge Stem Cell Institute, University of Cambridge, Cambridge CB2 0AW, UK; 3 Department of Pharmacology, University of Cambridge, Cambridge CB2 1QR, UK

**Keywords:** peer review, mentorship, scientific publishing, manuscript evaluation

## Abstract

Peer review is essential for maintaining the quality of published research, but this skill is often not explicitly taught to early career researchers (ECRs). Joint peer review with supervisors can both promote ECR professional growth and enhance review quality. We describe the advantages and best practices of joint peer review.

## Strengths and Weaknesses of the Peer Review Process

Peer review is the process of subjecting a manuscript to the inspection of experts in the same field in order to increase the value of the work for the broader scientific community [1]. Although it is sometimes criticised for being slow and subject to bias, it is essential for identifying mistakes, ensuring that conclusions are robustly supported by evidence [2], and ideally also for identifying reasonable experiments whose outcome would strengthen the study. A good review should provide feedback that is objectively fair, reasonable, and constructive even if it is not necessarily positive [3]. To help ensure this is the case, it is becoming more common for journals to publish a record of reviewer comments and author responses, and for reviewers to sign their names to their comments for the sake of transparency [4]. Even if performed in a blinded fashion, it’s good practice to treat reviews as if they would be published. Despite its importance, carrying out quality peer review is time-consuming and uncompensated, leading to lower than desirable levels of engagement in peer review, especially among senior academics who are best placed to provide expertise [5,6]. Collaborative peer review between a supervisor and an ECR (e.g. graduate students and postdocs), offers a potentially mutually beneficial solution to this challenge, since it provides hands-on training for the ECR and often increases the quality of the resulting review [7–9]. Many journals permit joint peer review when asked, and some (e.g. *Nature Communications, the Journal of Biological Chemistry*, and *FASEB Journal)* explicitly permit co-peer review in recognition of the widespread nature of this practice [8,10–12]. Indeed, surveys of peer review experiences across different institutions around the world found that nearly 80% of responding ECRs had co-reviewed a manuscript with their supervisor [13,14]. While widespread joint reviewing seems positive, it must be done properly. If ECRs do the bulk of the work without proper guidance or acknowledgement (ghostwriting), the quality of the review can suffer and ECRs do not benefit from training and career development opportunities [15]. Indeed almost half of survey respondents had not received recognition for their contribution [13]. To address this issue, ECRs and supervisors can advocate to journals for proper recognition [7], and ECRs can identify other mentors and resources (see Table 1) to enable joint peer review, as discussed in greater detail below.

## The joint peer review process

**Figure 1:**
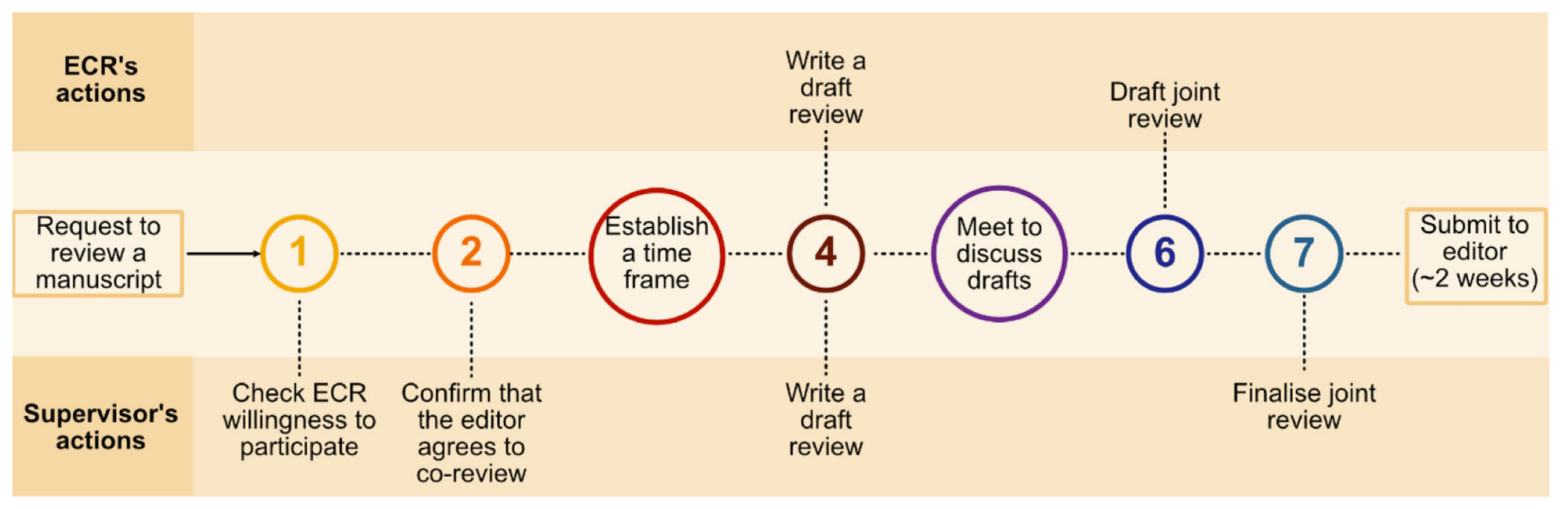
The process of joint peer reviewing. After confirming with the editor, the supervisor should meet with an ECR well-versed in the subject matter to establish a time frame and discuss the steps for a joint peer review. Both ECR and supervisor should then independently read the manuscript and draft their own review prior to meeting to discuss their suggestions. Finally, these drafts are combined to reflect the consensus opinions of the ECR and supervisor before being finalised and submitted to the journal.

The process of conducting a joint peer review often begins when a supervisor or mentor is approached by a journal to review a manuscript, and they know of an ECR who has relevant subject matter expertise in the area of the manuscript. They can then approach the ECR to ask if they are interested in a joint review and can commit to submitting the review within the necessary time frame (Figure 1). The supervisor should also confirm that the ECR understands the confidentiality of the review process and also confirm with the editor that a joint review with an ECR is permissible, and that the ECR will receive appropriate credit for their contribution.

At this point, a timeframe for reviewing the manuscript should be established. For example, if the review is due in 14 days, the supervisor and ECR should schedule a time to meet to discuss the manuscript, perhaps a week before the due date. Prior to that meeting, both reviewers should independently read the manuscript and draft reviews which should include a summary of the manuscript, major comments, minor comments, and notes to the editor. Once both drafts are independently completed, they should be shared and then discussed openly to identify areas of agreement, clarify questions and areas of disagreement, and further develop thinking behind suggestions. These discussions will reveal major and minor points to include in a final review, and how best to combine the two separate documents into a coherent whole to be proofread and polished before submission. The supervisor and ECR can then agree on whether to request their names to be published with the review or kept anonymous.

## Benefits to the ECR

Participating in peer review offers numerous benefits to ECRs, as testified by ECRs from the Young Investigators program of the European Journal of Pharmaceutics and Biopharmaceutics [9] and almost all (95%) surveyed ECRs who indicated it is “a beneficial training exercise” [13]. These benefits include:

- While resources on peer review are available (Table 1), these skills are often not formally taught. Engaging in joint peer review with a more experienced supervisor can provide ECRs with the necessary confidence to comment constructively on the work of others [16]. Indeed, this experience can build transferable critical thinking skills by encouraging ECRs to think deeply about experimental design and data analysis which in turn can improve their own manuscript and grant writing [17].
- Understanding what makes a manuscript interesting to a broad audience and compelling to an editor and reviewer will inevitably improve the quality of the ECR’s own manuscripts and grant applications, potentially leading to better-composed studies more likely to be published or funded. While some of these skills are acquired by reading the literature or discussing papers in journal clubs, the active process of peer review demands a higher level of scrutiny that teaches ECRs about standards and expectations of their field.
- Peer review enables ECRs and supervisors to stay abreast of the latest developments in the field, since submitted studies are by definition unpublished and may not yet have been deposited on preprint servers.
- Participating in joint peer review gives ECRs access to responses of other reviewers to complement those jointly developed with their supervisor. These other reviews can provide a confidence-building ‘sense-check’ that the same key issues were caught by others, while also illuminating issues they missed that were noticed by their supervisor or by other reviewers. Understanding how editors and journals respond to these comments provides valuable insight into the publishing process and allows ECRs to start building valuable relationships that will serve them well as they seek to publish their own studies.
- Participation in peer review is a way to demonstrate active engagement with the community and can be included in a *Curriculum Vitae* when seeking a position.
- Peer reviewing manuscripts exposes the ECR to alternative career paths such as scientific writing and publishing.

Should the supervisor not be willing to take the time to help train the ECR, they may be able to turn to other mentors with subject matter expertise, and/or to resources to provide formal training or mentorship, some of which are listed in Table 1.

## Opportunities and challenges for the supervisor

Ideally, supervisors or mentors with subject matter expertise would frequently peer review manuscripts, but in reality their many other responsibilities often mean they can do so only selectively. One of these other responsibilities is supporting the career development of ECRs in their groups, so joint peer review can enable them to provide a service to the community while also helping their ECR develop valuable skills. If the ECR already has a strong grasp of experimental design and understands the field well enough to understand the strengths and weaknesses of different technical approaches, they will be able to provide both big-picture and nuanced technical feedback on the manuscript. In this scenario, the total time taken by the supervisor to develop the joint review may not be much more than it would have taken them to write it themselves, and sharing thoughts with the ECR would likely lead to insights on both sides. Often, the ECR is still developing these skills and will require more active support from the supervisor. In this scenario, the supervisor will take time to explain their reasoning where they identified issues that the ECR has not, in some cases by sharing other relevant literature with them. While more time-consuming for the supervisor, this can be an effective way to provide hands-on training outside of the normal sphere of interactions. It’s important to assess whether the ECR understands the science well enough to provide constructive criticism prior to asking them to jointly review a manuscript, for example by monitoring their contributions to critical discussion of manuscripts in ‘journal clubs’. In summary, bringing the ECR into the process is a valuable training opportunity that ideally would spark new ideas, diversify points of view, and provide additional technical feedback to generate a more comprehensive review than would have been generated by the supervisor working alone [7–9].

## Benefits to the peer review process

When two individuals independently identify the same concern, it increases confidence in the validity of that concern. This is particularly important given the potential for individual biases or oversights in the peer review process [18]. Different reviewers often notice different aspects of a manuscript, leading to a more thorough and complete review when thoughts are combined. This is particularly relevant when formulating specific experiments that could be performed to strengthen a manuscript, since discussing it jointly can ensure that the suggestion is reasonable and likely to substantially improve the quality of the evidence to support (or test) a claim the authors wish to make. Having two sets of eyes on the technical aspects of the manuscript may also support scientific integrity by helping catch omitted details or flaws in experimental design or data analysis. Should there be disagreement between the ECR and supervisor, openly discussing it is the best approach to understanding and resolving the disagreement and producing a balanced and cohesive joint review. Peer review may also benefit editors and publishers by ensuring that the next generation of scientists is well trained in peer review and perhaps also since senior scientists are more likely to accept an offer to review, though they should remain vigilant about the potential of ghostwriting. In summary, joint peer review offers multiple benefits to the scientific community when performed appropriately, and we encourage its practice.

## Figures and Tables

**Table 1:** List of resources for peer review, and courses and programmes that include a peer review component.

Name	Description	Reference
Peer review- Why, when and how	General guide to peer reviewing	[1]
Peer Review in Scientific Publications: Benefits, Critiques, & A Survival Guide	Overview of the peer review process	[2]
Reviewer Hub	Compilation of tools, information, and guidance	https://www.elsevier.com/reviewer
Certified Peer Reviewer Course	Online course in peer review by Elsevier	https://researcheracademy.elsevier.com/navigating-peer-review/certified-peer-reviewer-course
Nature Masterclass “Focus on Peer Review”	Peer review course	https://masterclasses.nature.com/online-course-on-peer-review/16507836
Early Career Reviewer Program	Grant peer review programme at the National Institutes of Health’s (NIH) Center for Scientific Review for new independent investigators	https://public.csr.nih.gov/ForReviewers/BecomeAReviewer/ECR
Reviewer mentoring programme	Journal of Neuroscience program for one-on-one training in peer review for graduate students and postdocs	https://www.jneurosci.org/content/38/3/511
Early Career Researcher Reviewer Mentoring Programme	Peer review mentoring program for postdocs provided by the British Psychological Society and Wiley publishers	https://bpspsychub.onlinelibrary.wiley.com/hub/earlycareermentoring
Young Investigators program of the European Journal of Pharmaceutics and Biopharmaceutics	Program for early-career researchers that includes an introduction to peer review	[9]
